# Predicting Severe Enterovirus 71-Infected Hand, Foot, and Mouth Disease: Cytokines and Chemokines

**DOI:** 10.1155/2020/9273241

**Published:** 2020-01-31

**Authors:** Weijian Zhang, Zhigang Huang, Mingyuan Huang, Jincheng Zeng

**Affiliations:** ^1^Dongguan Key Laboratory of Environmental Medicine, School of Public Health, Guangdong Medical University, Dongguan 523808, China; ^2^Dongguan Key Laboratory of Medical Bioactive Molecular Developmental and Translational Research, Guangdong Provincial Key Laboratory of Medical Molecular Diagnostics, Guangdong Medical University, Dongguan 523808, China

## Abstract

Enterovirus 71 (EV71) is one of the most common intestinal virus that causes hand, foot, and mouth disease (HFMD) in infants and young children (mostly ≤5 years of age). Generally, children with EV71-infected HFMD have mild symptoms that resolve spontaneously within 7-14 days without complications. However, some EV71-infected HFMD cases lead to severe complications such as aseptic meningitis, encephalitis, acute flaccid paralysis, pulmonary edema, cardiorespiratory complication, circulatory disorders, poliomyelitis-like paralysis, myocarditis, meningoencephalitis, neonatal sepsis, and even death. The mechanism of EV71 pathogenesis has been studied extensively, and the regulation of host immune responses is suspected to aggravate EV71-induced severe complications. Recently, several cytokines or chemokines such as TNF-*α*, IFN-*γ*, IL-1*β*, IL-18, IL-33, IL-37, IL-4, IL-13, IL-6, IL-12, IL-23, IL-27, IL-35, IL-10, IL-22, IL-17F, IL-8, IP-10, MCP-1, G-CSF, and HMGB1 have been reported to be associated with severe EV71 infection by numerous research teams, including our own. This review is aimed at summarizing the pathophysiology of the cytokines and chemokines with severe EV71 infection.

## 1. Introduction

Enterovirus 71 (EV71) is one of the most common and neurotropic intestinal virus that causes hand, foot, and mouth disease (HFMD) in infants and young children (mostly ≤5 years of age). EV71-infected HFMD usually starts with blisters and rashes in the mouth or on the hands and feet [1]. Generally, children with HFMD have mild symptoms that resolve spontaneously within 7-14 days without complications. However, some EV71-infected HFMD cases can have serious complications such as aseptic meningitis, encephalitis, acute flaccid paralysis, pulmonary edema, cardiorespiratory complication, circulatory disorders, poliomyelitis-like paralysis, myocarditis, meningoencephalitis, and neonatal sepsis. Especially, brainstem encephalitis, neurogenic pulmonary edema, or cardiopulmonary failure are the main causes of death [2]. Between 1969 and 1972, Schmidt et al. first isolated EV71 from the brain of a fatal case of encephalitis and confirmed by comparison of EV71 which isolated from the original clinical faecal specimens in the central nervous system patients with encephalitis or aseptic meningitis [3]. These results suggest that HFMD infected with EV71 can cause encephalitis. From April to August 1997, EV71 epidemic occurred for the first time in Sarawak, Malaysia, causing at least 31 children to die of viral myocarditis, a severe form of HFMD [4, 5]. It also became the first large-scale HFMD outbreak in the Asia-Pacific region. Besides, a total of 405 severe cases (including 78 deaths) were reported during the widespread HFMD epidemic in Taiwan, China, in 1998 [6, 7]. The infection rates of EV71 in severe cases and death cases were 75% and 92%, respectively. What is more, the complications included encephalitis, aseptic meningitis, pulmonary edema or hemorrhage, acute flaccid paralysis, and myocarditis, and the main cause of death was pulmonary edema or pulmonary hemorrhage. Homoplastically, there were 11 severe EV71-infected HFMD cases with neurologic complications, such as brainstem encephalitis and aseptic meningitis, and 3 children died from a combination of acute pulmonary edema and heart and respiratory failure in Shandong, China, in 2007 [8].

**Figure 1:**
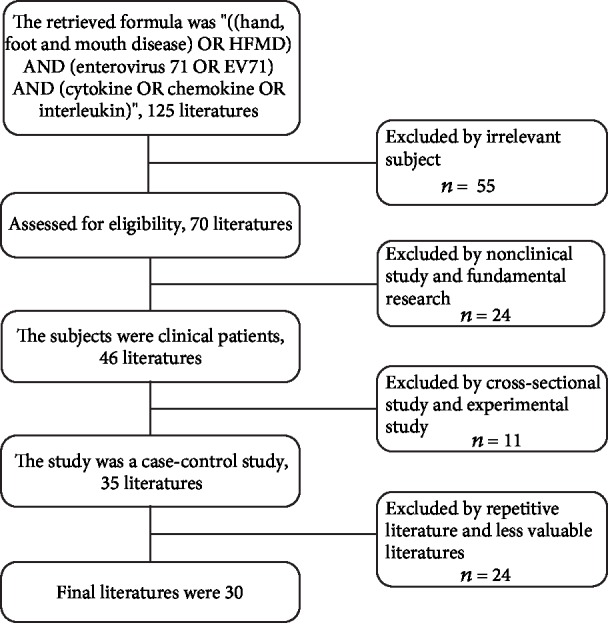
Study design and retrieval strategy.

However, the mechanism of severe complications caused by EV71 is still unclear. The infection of EV71 depends on multiple effects of the virus, host, and environment. In particular, genes involved in mediating EV71 virus escape from host intrinsic or adaptive immune response monitoring are closely related to EV71 susceptibility [9]. In this process, cytokines and chemokines that bridge the innate and adaptive immune responses of the body become targets for screening EV71 infection-related genetic susceptibility genes. For example, IL-6 and MCP-1 genes may affect the risk and severity of EV71 infection by affecting their gene expression and regulating inflammatory response. Most importantly, the level of cytokines fluctuates a lot between healthy volunteers, mild cases, and severe EV7-infected HFMD with complications, which indicates that cytokines may play a critical role in the progress of EV71 infection and may become targets of diagnosis and treatment. Therefore, we are carrying out a review according to study design and retrieval strategy of Figure 1 to illustrate the cytokine storm with severe EV71 infection.

## 2. TNF-*α*

TNF-*α* is mainly secreted by mononuclear macrophages. Besides killing tumor cells, TNF-*α* is also involved in immune regulation, fever, and inflammation. TNF-*α* involves the occurrence and development of many diseases, which can promote the production of various inflammatory factors by T cells to promote the occurrence of inflammatory response. The serum levels of TNF-*α* in severe EV71-infected patients were significantly higher than those in mild patients [10–12] (shown as Table 1), which indicated that TNF-*α* may be involved in disease severity. High levels of TNF-*α* were detected in severe patients with complications (including brainstem encephalitis, neurogenic pulmonary edema, and sepsis) at disease onset, and then decreased with disease progression [10]. The critical HFMD group (frequent convulsions, coma, brain hernia, pulmonary rales, or circulatory insufficiency) also had higher levels of TNF-*α* than the severe group (acute flaccid paralysis or convulsions) and mild group [11]. Therefore, TNF-*α* is a crucial cytokine in severe EV71-infected HFMD.

## 3. IFN-*γ*

IFN-*γ* is produced by activated T cells, natural killer cells (NK cells), and NKT cells. IFN-*γ* is a hallmark cytokine of type I helper T cells (Th1 cells), which have antivirus, immune regulation, and antitumor properties [13]. Studies suggested that the enhanced expression of IFN-*γ* is associated with life-threatening complications in severe EV71-infected HFMD cases [12, 14–17]. However, a study showed that the changes of the serum levels of IFN-*γ* in severe patients were different, which the levels of IFN-*γ* were very low in the first day and then increased for the first three days and then decreased [10] (as seen in Table 1). Sun et al. [12] divided severe HFMD patients into two groups including central nervous system disease (CNSD) group and neurogenic pulmonary edema (NPE) group. Interestingly, serum IFN-*γ* levels were higher in the NPE group than those in the CNSD group, and the differences were statistically significant (*p* < 0.01). The polymorphisms of IFN-*γ*+874 A allele was observed with significantly greater frequency in 65 Chinese patients with EV71-infected encephalitis (76.2%) compared with HFMD patients without complications (61.1%, *p* < 0.01, [15]), which implying that IFN-*γ*+874 A allele is associated with susceptibility to EV71 encephalitis in Chinese patients. Moreover, the serum levels of IFN-*γ* elevated a lot in patients with brainstem encephalitis and pulmonary edema [17], which suggests that IFN-*γ* may be involved in the progress of EV71-caused severe complications.

## 4. IL-1 Family

### 4.1. IL-1*β*

IL-1*β* is a proinflammatory cytokine mainly produced by monocytes, endothelial cells, and fibroblasts in response to infection. When the local concentration is low, IL-1*β* can costimulate antigen-presenting cells (APCs), activate T cells, promote the proliferation of B cells, and secrete antibodies for immune regulation [18]. As shown in Table 1, during the time of hospitalization, patients with EV71-infected complications (encephalitis and cardiorespiratory compromise) had elevated levels of IL-1*β* compared with those with aseptic meningitis and acute flaccid paralysis [14]. Evidence from other diseases supports the putative importance of the elevated IL-1*β* response in the periphery contributing to EV71-associated cardiac failure. For example, elevated IL-1*β* level in the lungs is linked to acute respiratory distress syndrome [19]. In addition, the plasma levels of IL-1*β* in severe cases and critical cases (including coma with cerebral hernia, respiratory failure, or circulatory collapse) were significantly higher than those in mild and normal patients at acute stage, and then declined in convalescence [20]. In summary, IL-1*β* may play an indispensable role in the pathogenesis of EV71-infected HFMD with severe complications.

### 4.2. IL-18

IL-18, a potent proinflammatory cytokine, can be produced by a variety of tissue cells, which can induce Th1 cells to produce other cytokines, to active NK cells' cytotoxic activity and to promote T cell proliferation. IL-18 plays an important role in the occurrence and development of diabetic nephropathy (DN) [21]. Similarly, the serum levels of IL-18 significantly elevated in EV71-infected HFMD patients (*p* < 0.001), especially those with pulmonary edema, gastrointestinal symptoms (e.g., vomiting and diarrhea), and myocardial injury [22]. In addition, IL-18 peaked slightly on day 3 and maximally on day 11 in patients with severe HFMD, which suggested that cytokine levels of IL-18 may be useful prognostic indicators of HFMD severity, potentially related to immune impairment caused by EV71 infection.

### 4.3. IL-33

IL-33 is a key cytokine involved in type 2 immunity and released upon cell necrosis and drives inflammation as a damage-associated molecular pattern. IL-33 can bind to ST2, a member of the IL-1 receptor family, activates NF-*κβ* and MAPK, promotes the production of Th2 cytokines, and participates in allergy [23]. A study found that a marked increase in levels of IL-33 in severe patients (including myoclonus, vomiting, ataxia, irritability, and hypersomnia) and critical cases (quickly developed acute respiratory failure and PE) presenting with neurological manifestations compared to mild patients [24].

### 4.4. IL-37

IL-37, a novel member of the IL-1 family, is an inhibitor of innate and adaptive immunity and can inhibit the expression of a variety of inflammatory factors [25]. IL-37, mainly expressed in dendritic cells, monocytes, and plasma cells after TIR ligand activation, inhibits inflammatory cytokines and augments the level of anti-inflammatory IL-10. It has been studied that high levels of IL-37 can suppress inflammatory responses and clinical signs in various autoimmune diseases, such as rheumatoid arthritis, ankylosing spondylitis, and systemic lupus erythematosus [26]. Importantly, they were twofold higher in IL-37 levels in HFMD patients with typical symptom than that in control subjects [27].

## 5. IL-2 Family

### 5.1. IL-4

IL-4 is mainly produced by Th2 cells, mast cells, and basophils, which can promote the proliferation and differentiation of B cells, induce IgG1 and IgE production, facilitate Th0 cells to differentiate into Th2 cells, and so on [28]. The dynamic changes of the IL-4 with the progression of HFMD and its severity have been studied [10, 22, 29]. The levels of IL-4 among the cases were significantly different and increased from the 2nd day to the 4th day (*p* < 0.001), especially in the fatal cases accompanied with brainstem encephalitis, neurogenic pulmonary edema, and sepsis [10]. On the other hand, serum levels of IL-4 were significantly higher in the EV71-infected encephalitis patients than those in the HFMD-alone patients when adjusting for age and sex [29]. In this way, it is strongly believed that EV71 infection is associated with higher risk of encephalitis development.

### 5.2. IL-13

IL-13 is produced by Th2 cells, which can induce mononuclear cell differentiation, enhance the expression of MHC II molecules, inhibit the secretion of LPS-induced mononuclear factors, and control the inflammatory response [30]. In EV71-causing severe HFMD, IL-13 plays a potential anti-inflammatory activity that an exaggerated production of IL-13 was observed in patients and usually peaked during the early phase of hospitalization [17]. More significantly, IL-13 levels were consistently elevated in the pulmonary edema group, which considered that IL-13 might contribute to the pathogenesis of PE. Our previous study [31] also found that the serum of IL-13 levels of clinical stage IV (cardiopulmonary failure) in EV71-infected HFMD patients were correspondingly about two times higher than those of clinical stage II (early cardiopulmonary failure) in EV71-infected HFMD patients in the day of admission. However, it remains controversial that a study [32] declared that the G allele at the rs20541 locus of IL-13 gene was not a risk factor for EV71-infected severe HFMD in either male or female patients.

## 6. IL-6/IL-12 Family

### 6.1. IL-6

IL-6 is mainly produced by mononuclear macrophages, Th2 cells, vascular endothelial cells, and fibroblasts. It can stimulate the proliferation of activated B cells and then secrete antibodies, stimulate T cell proliferation as well as the synthesis of acute phase proteins, so that it can participate in inflammatory response. As shown in Table 1, many studies [10, 11, 31, 33–35] have reported the vital role of high levels of IL-6 in EV71-infected patients with severe complications. The CSF levels of IL-6 in study patients were found to be consistently higher during the first 2 days of central nervous system (CNS) involvement (including encephalitis, poliomyelitis-like syndrome, meningitis, and pulmonary edema) than afterward [33]. Similarly, we also found a high expression of IL-6 levels on EV71-infected HFMD patients, especially in clinical stage IV (cardiopulmonary failure) [31]. Lee et al. [35] considered the elevated IL-6 levels in EV71-induced aseptic meningitis as importance for IL-6 had a strong association with aseptic meningitis and the cutoff value for IL-6 was 66 pg/mL according to the ROC analysis, which suggested that IL-6 may be an indicator of aseptic meningitis. Besides, IL-6-572 G allele may increase the risk of EV71 encephalitis in that IL-6-572 G allele was significantly correlated with the susceptibility of Han Chinese patients to EV71 encephalitis [34]. Actually, IL-6 is a marker of nonspecific pathogen infection or inflammation. Thus, the rise of this marker levels only in a proportion of patients especially in severe patients.

### 6.2. IL-12

IL-12 is mainly produced by B cells and macrophages, which can stimulate the proliferation of activated T cells and promote the differentiation of Th0 cells into Th1 cells [36]. It also can induce the cytotoxic activity of NK cells and promote their secretion of cytokines such as IFN-*γ*, TNF-*α*, and GM-CSF. The levels of IL-12 in patients with severe EV71 HFMD (including encephalitis and pulmonary edema) were higher than those in patients with mild HFMD or the control group [16]. Furthermore, in severe HFMD, the levels of IL-12 in patients with encephalitis plus pulmonary edema were higher than those with encephalitis alone.

### 6.3. IL-23

IL-23 can promote the proliferation of T cells and the production of IFN-*γ* and induce the proliferation of memory T cells. It was reported that the serum levels of IL-23 were significantly higher in the viral encephalitis patients compared with HFMD-alone patients (*p* = 0.002, [29]). After adjustment for age and sex, elevated levels of IL-23 remained to be significantly associated with encephalitis, which might further play roles in the encephalitis following EV71 infection.

### 6.4. IL-27

IL-27 is produced by the activation of antigen-presenting cells in the early stage, promoting the proliferation of naive T cells, and cooperating with IL-12 to stimulate the production of IFN-*γ* in T cells and promoting the early Th1 cells [47]. Our previous study [44] found that serum IL-27 levels were distinctly higher in clinical stage III (early cardiopulmonary failure) EV71-infected HFMD patients than in clinical stage II (involvement of the nervous system) or clinical stage IV (cardiopulmonary failure) EV71-infected patients, which suggested that IL-27 may play a role in HFMD caused by EV71 infection, especially in patients with early cardiopulmonary failure.

### 6.5. IL-35

IL-35 is a member of the newly discovered IL-12 cytokine family, consists of an IL-12 subunit *α* chain (P35) and IL-27 subunit Epstein-Barr virus-induced gene 3 (EBI3) *β* chain. It is secreted not only by regulatory T (Treg) cells but also by CD8+ Treg cells, activated dendritic cells, and regulatory B cells. Treg cells and their secreted cytokines are currently believed to have a long-lasting immune tolerance effect, thus suggesting that IL-35 plays an important role in the immune tolerance period of viral infection [48]. In a recent study, an imbalance between Tregs and Th17 cells was observed in children with severe EV71-indcued HFMD (including cardiorespiratory complication) [45]. IL-35-secreting Tregs were also declined in patients with severe HFMD, and this observation was positively correlated with the Treg-to-Th17 cell ratio, which may play a key role in the pathogenesis of EV71-infected HFMD.

## 7. IL-10 Family

### 7.1. IL-10

IL-10, a well-known anti-inflammatory cytokine, is mainly produced by Th2 cells and mononuclear macrophages, which can inhibit the production of pro-inflammatory cytokines, for example, IL-10 suppresses T cells to synthesize IL-2, IFN-*γ*, but it can promote the differentiation and proliferation of B cells [49]. As seen in Table 1, IL-10 can be modulated in several acute and chronic neuropathological conditions [12, 15, 17, 31, 40]. The plasma levels of IL-10 in EV71-infection with pulmonary edema were very higher than those in nervous system dysregulation and brainstem encephalitis group [17], suggesting that increased IL-10 may have a protective effect in the development of PE by influencing the pulmonary capillary permeability. On the other hand, the IL-10-592 C allele was observed with higher frequency with critical EV71 infection (70.59%) compared with severe EV71 infection (41.43%, *p* < 0.01) and mild EV71 infection (43.81%, *p* < 0.01) [40], while IL-10-1082 A allele has greater frequency with EV71 encephalitis (86.2%) compared with HFMD patients without complications (77.0%, *p* < 0.05, [15]).

### 7.2. IL-22

IL-22 is mainly produced by acute phase proteins that promote inflammation and expressed by many immune cells, including innate and adaptive immune cells such as Th22, Th17, CD8+T, and dendritic cells (DCs) [50]. The plasma IL-22 levels as well as the expression levels of IL-22 mRNA were significantly higher in the EV71 severe cases than those in the EV71 mild cases [43]. Additionally, the plasma levels of IL-22 were positively correlated with the frequencies of cTh22 cells in the mild and severe EV71 infection HFMD, which indicated that increased IL-22 levels might be predominantly secreted by cTh22 cells in the patients with EV71-infected HFMD.

## 8. IL-17 Family

IL-17, the main effector of Th17 cells, is secreted by CD4+ T cells and can induce epithelial cells, endothelial cells, and fibroblasts to synthesize and secrete IL-6, IL-8, and G-CSF [51]. IL-17F is one of the six ligands in the IL-17 family, which can promote the release of proinflammatory cytokines to modulate inflammatory response. The gene polymorphism of IL-17F with EV71 encephalitis in HFMD has been studied. One study [41] demonstrated that the frequency of IL-17F-7488 C allele was significantly lower among the patients with EV71 encephalitis (5.2%) as compared to that without complications (15%, *p* = 0.006). However, the other study [42] showed that the serum IL-17F levels in rs1889570 T/T and T allele were significantly elevated in EV71 encephalitis cases. In a word, IL-17F gene polymorphisms are associated with the susceptibility to severe EV71 infection.

## 9. Chemokines

### 9.1. IL-8

IL-8 is a chemokine secreted by macrophages and other cell types, and the main biological activity is to attract and activate neutrophils so that it could produce local inflammation in the body. The level of IL-8 in patients with BE or PE at admission is significantly elevated, and in the BE+PE group, the levels were maximal [39]. In the BE+PE group, high IL-8 levels lasted longer than normal compared to other groups with milder cases. A recent report [38] showed that IL-8 exhibited high AUC values (0.95) through the ROC curve assay and the serum levels of IL-8 in EV71 critical infection (including frequent convulsions, coma, brain hernia, and pulmonary rales) were highest compared with mild cases and severe cases. Both Li et al. [52] and Xu et al. [37] considered that the frequency of IL-8-251 T alleles among the severe cases (such as encephalitis and respiratory failure) was significantly higher than that of mild cases. In summary, the studies indicated a strong correlation between IL-8 and EV71-infected HFMD severity.

### 9.2. IP-10

IP-10, a chemokine belonged to CXC family, is mainly secreted by monocytes, dendritic cells, NK cells, and other cells stimulated by IFN-*γ*. IP-10 can mediate Th1 inflammatory response [53]. In EV71-infected HFMD patients, the levels of IP-10 were significantly elevated as compared to healthy controls [24]. The association between IP-10 polymorphism in children and EV71infection has been studied [46]. The result showed that the frequency of carrying CT+TT genotype (10.3) and T allele (6.0%) in EV71-infected cases was significantly lower than that of the controls (29.2 and 15.6%), which indicated that IP-10-1596 T allele may be a beneficial factor for EV71 infection.

### 9.3. MCP-1 and RANTES

MCP-1, a chemokine belonging to CC family, is an important proinflammatory cytokine, which can be secreted and produced by monocytes, macrophages, fibroblasts, and other cells. In addition, MCP-1 has a specific chemotactic activation effect on monocytes/macrophages. The main functions of RANTES are specific chemotactic T cells, monocytes and eosinophils, which play an important role in the activation of killer T cells in the immune response. The MCP-1 and RANTES levels with EV71 severity (mild vs. severe groups) and complications (E vs. E+P groups) in children were significantly elevated [16], which suggested that MCP-1 and RANTES participate in EV71-infected HFMD pathogenesis, and could be of potential value in monitoring disease progression and predicting prognosis.

## 10. G-CSF

Granulocyte colony-stimulating factor (G-CSF) is mainly produced by endotoxin, TNF-*α*, and IFN-*γ*-activated monocytes and macrophages, and it can act on the proliferation, differentiation, and activation of hematopoietic cells of the neutrophil lineage [54]. The levels of G-CSF were significantly increased in plasma from very severe EV71-infected patients presenting with acute respiratory failure [24]. For patients with neurological manifestations, G-CSF levels were remarkably higher in the cerebrospinal fluid than in plasma [14], suggesting that they may be predominant mediators induced when neurological damage occurs in the cerebrospinal fluid.

## 11. HMGB1

HMGB1 is a highly conserved nuclear protein that is ubiquitous in mammalian cells. During the process of infection and inflammation, activated mononuclear macrophages or necrotic cells can release a large amount of HMGB1, which can induce the production of TNF-*α* and IL-6 and other proinflammatory factors [55]. It has been found that serum HMGB1 was significantly increased in EV71-infected HFMD during hospitalization, especially in the severe and critical HMFD groups [11], but it declined during the recovery phase. Furthermore, HMGB1 level was positively correlated with the alteration of serum IL-6 and TNF-*α* concentrations. Therefore, HMGB1 could be taken as an indicator for the severity of EV71-infected HMFD.

## 12. Conclusion

The change of cytokine concentration is of great significance for disease prevention, diagnosis, and treatment. In recent years, more and more studies have been conducted on the cytokine levels related to severe complications caused by EV71 infection, suggesting that the important role of cytokines in the occurrence and development of EV71 infection has been recognized. After EV71 infection, susceptible cells and nonspecific immune cells are stimulated first to produce cytokines such as TNF-*α*, IFN-*γ*, and IL-6. These cytokines play an important role in the early control of viral replication and infection. Furthermore, the activation of these cells by cytokines leads to the secretion of inflammatory mediators and cytokines, interferes with viral replication, and kills virus-infected host cells. In this review, several cytokines and chemokines such as TNF-*α*, IFN-*γ*, IL-1*β*, IL-18, IL-33, IL-37, IL-4, IL-13, IL-6, IL-12, IL-23, IL-27, IL-35, IL-10, IL-22, IL-17F, IL-8, IP-10, MCP-1, G-CSF, and HMGB1 that were screened in recent years were closely related to severe EV71 infection. Therefore, analysis of the levels of these cytokines and chemokines in severe EV71 infection is of great value for early prevention of childhood infection and reduction of regional EV71 infection rate. However, existing studies need to continue to increase the sample size and conduct studies in different regions of the population, so as to further the study of cytokines and chemokine related to severe EV71 infection and provide evidence for revealing the important role of cytokines and chemokine in the occurrence and development of diseases.

## Figures and Tables

**Table 1:** The association between cytokines, chemokines, and EV71-infected HFMD.

Cytokines/chemokines (sources)	Countries/regions	Study type	Sample type	Complications/severe cases	OR/ROC	Level/value	Progression	*p* value	Study (year)
TNF-*α* (M*φ*)	China-Zhengzhou	Case-control	Serum	Fatal cases (*n* = 50): BE+NPE+sepsis	4.325	Peak value Declined	The 2nd day 3-5 days	0.003 < 0.001	Duan et al. (2014) [10]
	China-Shandong	Case-control	Serum	NPE (*n* = 37), CNSF (*n* = 29)	4.593	Elevated	The 3rd day	0.001	Sun et al. (2018) [12]
	China-Zhejiang	Case-control	Serum	Severe (*n* = 40): AFP, convulsions Critical (*n* = 12): coma, dyspnea, PE	—	Elevated	Hospitalized	<0.01	Zheng et al. (2017) [11]

IFN-*γ* (Th1)	China-Zhengzhou	Case-control	Serum	Fatal cases (*n* = 50): BE+NPE+sepsis	4.325	Low High	The 2nd day 3-5days	0.017 <0.017	Duan et al. (2014) [10]
	China-Shandong	Case-control	Serum	NPE (*n* = 37) CNSF (*n* = 29)	4.593	Elevated Declined	The 1st day The 3rd day	<0.01	Sun et al. (2018) [12]
	China-Qingdao	Case-control	Blood	Encephalitis (*n* = 65)	2.04	(A allele) Elevated	—	<0.01	Yang et al. (2012) [15]
	Taiwan	Case-control	Plasma	ANSD (*n* = 25), PE (*n* = 14), BE (*n* = 34)	—	Elevated	—	0.048	Wang et al. (2003) [17]

IL-1*β* (M*φ*)	Malaysian	Case-control	Serum, CSF	AM (*n* = 8), E (*n* = 21), AFP (*n* = 1), CRC (*n* = 11)	—	Elevated	Hospitalized	0.013	Griffiths et al. (2012) [14]
	China-Nanjing	Case-control	Plasma, CSF	Severe (*n* = 25): myoclonus, ataxia Critical (*n* = 12): coma, RF, CC	2.168	Elevated Declined	Acute stage Convalescence	<0.05 <0.05	Ye et al. (2015) [20]

IL-6 (Th2, M*φ*)	Taiwan	Case-control	CSF, serum	E (12), PLS (3), AM (7), PE (8)	—	Elevated	The 2nd day	<0.001	Lin et al. (2003) [33]
	China-Zhengzhou	Case-control	Serum	Fatal cases (*n* = 50): BE+NPE+sepsis	4.325	Elevated Declined	2-3 days 4-5 days	0.003 <0.001	Duan et al. (2014) [10]
	China-Zhejiang	Case-control	Serum	Severe (*n* = 40): AFP, convulsions Critical (*n* = 12): coma, dyspnea, PE	—	Elevated Declined	Hospitalized convalescence	<0.01 <0.01	Zheng et al. (2017) [11]
	China-Guangdong	Case-control	Serum	Stages II (*n* = 30), III (*n* = 30), IV (*n* = 22)	—	Elevated	TP0	<0.01	Chen et al. (2014) [31]
	China-Qingdao	Case-control	CSF, serum	Encephalitis (*n* = 59)	3.31	(G allele) Elevated	—	<0.001	Yuan et al. (2015) [34]
	Korea, Seoul	Case-control	Serum	Aseptic meningitis (*n* = 54)	7.44/AUC = 0.95	Elevated	—	<0.001	Lee et al. (2018) [35]

IL-8 (M*φ*)	China-Shandong	Case-control	Plasma	Severe (*n* = 80): E (36) + CNSF + RF+ dysaemia	1.8	(T allele) Elevated	—	0.012	Xu et al. (2016) [37]
	China-Nanjing	Case-control	Plasma	Severe (*n* = 25): myoclonus, ataxia Critical (*n* = 12): coma, RF, CC	2.168	Elevated Declined	Acute stage Convalescence	<0.05 <0.05	Ye et al. (2015) [20]
	China-Shenzhen	Case-control	Serum	Severe (*n* = 41): CNSF+CRC Critical (*n* = 34): convulsions+coma +BH+dyspnea	AUC = 0.46	Elevated	—	<0.001	He et al. (2019) [38]
	Beijing	Case-control	Serum	Mild (*n* = 32): CNSF, RF Severe (*n* = 59): PE (8), GT (16), MI (4), death (4)	—	Peak value Peak value	The 6th day The 11th day	<0.001	Han et al. (2014) [22]
	Beijing	Case-control	Serum	BE (*n* = 47) PE (*n* = 12) BE+PE (*n* = 25)	5.833	Peak value Declined	The 1st day The 4th day	NS NS 0.002	Wang et al. (2014) [39]

IL-4 (Th2)	China-Zhengzhou	Case-control	Serum	Fatal cases (*n* = 50): BE+NPE+sepsis	4.325	Elevated	2-4 days	0.001	Duan et al. (2014) [10]
	China	Case-control	Serum	Viral encephalitis (*n* = 24)	15.721	Elevated	—	<0.001	Zhang et al. (2015) [29]
	Beijing	Case-control	Serum	Mild (*n* = 32): CNSF, RF Severe (*n* = 59): PE (8), GT (16), MI (4), death (4)	—	Higher Higher	2-6 days 7-14 days	<0.001	Han et al. (2014) [22]

IL-10 (Th2, M*φ*)	Taiwan	Case-control	Plasma	ANSD (*n* = 25), PE (*n* = 14), BE (*n* = 34)	—	Elevated	—	<0.001	Wang et al. (2003) [17]
	China-Shandong	Case-control	Serum	NPE (*n* = 37) CNSD (*n* = 29)	4.593	Elevated Declined	The 3rd day The 5th day	0.001 <0.05	Sun et al. (2018) [12]
	China-Qingdao	Case-control	Plasma	Severe (*n* = 70): vomiting, convulsion Critical (*n* = 17): coma, CRC, PE, CC	2.998	(C allele) Elevated	—	<0.01	Zhao et al. (2017) [40]
	China-Guangdong	Case-control	Serum	Stages II (*n* = 30), III (*n* = 30), IV (*n* = 22)	—	Elevated	TP0	<0.01	Chen et al. (2014) [31]
	China-Qingdao	Case-control	Blood	Encephalitis (*n* = 65)	1.86	(A allele) Elevated	—	<0.05	Yang et al. (2012) [15]

IL-12 (Th1)	China-Hangzhou	Case-control	Plasma	Mild (*n* = 11): typical symptom Severe cases (*n* = 28): E (17), E+PE (11)	—	Elevated	Within 24 h	<0.01	Shang et al. (2017) [16]

IL-13 (Th2)	Taiwan	Case-control	Plasma	ANSD (*n* = 25), PE (*n* = 14), BE (*n* = 34)	—	Elevated	—	0.048	Wang et al. (2003) [17]
	China-Guangdong	Case-control	Serum	Stages II (*n* = 30), III (*n* = 30), IV (*n* = 22)	—	Elevated	TP0	<0.01	Chen et al. (2014) [31]
	China-Dongying	Case-control	Serum	Severe (*n* = 162): vomiting, AFP, convulsions, etc.	0.969	(G allele) NS	—	>0.05	Zhang et al. (2019) [32]

IL-17F (Th17)	China-Qingdao	Case-control	Blood	Encephalitis (*n* = 58)	0.31	(C allele) Declined	—	0.006	Lv et al. (2013) [41]
	China-Qingdao	Case-control	Blood	Severe (*n* = 115): typical symptom encephalitis (*n* = 72)	1.916	(T allele) Elevated	—	0.001	Li et al. (2018) [42]

IL-18 (Th1)	Beijing	Case-control	Serum	Mild (*n* = 32): CNSF, RF Severe (*n* = 59): PE (8), GT (16), MI (4), death (4)	—	Peak value Peak value	The 3th day The 11th day	<0.001	Han et al. (2014) [22]

IL-22 (Th17, Th22)	China	Case-control	Serum	Viral encephalitis (*n* = 24)	8.28	Elevated	—	0.026	Zhang et al. (2015) [29]
	China-Zhejiang	Case-control	Plasma	Mild (*n* = 32): typical symptom Severe (*n* = 24): CRC, BH, MI, etc.	—	Elevated	—	<0.01 <0.001	Cui et al. (2017) [43]

IL-23 (DC, M*φ*)	China	Case-control	Serum	Viral encephalitis (*n* = 24)	4.564	Elevated	—	0.002	Zhang et al. (2015) [29]

IL-33 (DC, M*φ*)	Shenzhen	Case-control	Plasma	Severe (*n* = 23): myoclonus, vomiting Critical (*n* = 8): acute RF, PE	2.348	Elevated	Hospitalized	<0.01	Zhang et al. (2013) [24]

IL-27 (APC)	China-Guangzhou	Case-control	Serum	Stages II (*n* = 55), III (*n* = 42), IV (*n* = 30)	—	— Elevated Declined	TP0 TP1 TP2	— <0.05 <0.01	Huang et al. (2016) [44]

IL-35 (Treg)	China-Henan	Case-control	Serum	Mild (*n* = 30): typical symptom Severe (*n* = 17): CNSF, CRC	—	Declined	—	<0.0001	Huang et al. (2017) [45]

IL-37 (APC)	China-Hefei	Case-control	Blood	Patients (*n* = 60): typical symptom	—	Elevated	—	<0.05	Lv et al. (2019) [27]

IP-10 (M*φ*)	Shenzhen	Case-control	Plasma	Severe (*n* = 23): myoclonus, vomiting Critical (*n* = 8): acute RF, PE	2.348	Elevated	Hospitalized	<0.01	Zhang et al. (2013) [24]
	China-Qingdao	Case-control	Blood	Patients (*n* = 58): typical symptom	0.35	(T allele) Declined	—	<0.05	Yang et al. (2013) [46]

MCP-1 (M*φ*)	China-Hangzhou	Case-control	Plasma	Mild (*n* = 11): typical symptom Severe cases (*n* = 28): E (17), E + PE (11)	—	Elevated	Within 24 h	<0.01	Shang et al. (2017) [16]
	Shenzhen	Case-control	Plasma	Acute RF (*n* = 8)	2.348	Elevated	Hospitalized	<0.01	Zhang et al. (2013) [24]

RANTES (NK, CD8^+^)	China-Hangzhou	Case-control	Plasma	Mild (*n* = 11): typical symptom Severe cases (*n* = 28): E (17), E + PE (11)	—	Elevated	Within 24 h	<0.01	Shang et al. (2017) [16]

HMGB1 (M*φ*)	China-Zhejiang	Case-control	Serum	Severe (*n* = 40): AFP, convulsions Critical (*n* = 12): coma, dyspnea, PE	—	Elevated Declined	Hospitalized recovered	<0.01 <0.01	Zheng et al. (2017) [11]

G-CSF (EC, FB)	Malaysian	Case-control	CSF	AM (*n* = 8), E (*n* = 21), AFP (*n* = 1), CRC (*n* = 11)	—	Elevated	Hospitalized	<0.01	Griffiths et al. (2012) [14]

M*φ*: monocytes/macrophages; Th: helper T lymphocytes; DC: dendritic cells; APC: antigen-presenting cells; Treg cells: regulatory T cells; NK: natural killer cell; EC: endothelial cells; FB: fibroblasts; E: encephalitis; AM: aseptic meningitis; AFP: acute flaccid paralysis; NPE: neurogenic pulmonary edema; CNSF: central nervous system failure; CSF: cerebrospinal fluid; PLS: poliomyelitis-like syndrome; BE: brainstem encephalitis; PE: pulmonary edema; CRC: cardiorespiratory complication; RF: respiratory failure; CC: circulatory collapse; BH: brain hernia; GT: gastrointestinal symptoms; MI: myocardial injury; ANSD: autonomic nervous system dysregulation; AUC: area under the curve; IP-10: interferon-induced protein 10; MCP-1: monocyte chemoattractant protein-1; HMGB1: high mobility group protein 1; G-CSF: granulocyte colony-stimulating factor. Stage II means involvement of the nervous system; stage III means early cardiopulmonary failure; and stage IV means cardiopulmonary failure. TP0 means the day of admission; TP1 means the day the disease improved; and TP3 means the day the disease recovered.
